# ‘*This is hardcore’*: a qualitative study exploring service users’ experiences of Heroin-Assisted Treatment (HAT) in Middlesbrough, England

**DOI:** 10.1186/s12954-023-00785-y

**Published:** 2023-05-12

**Authors:** Fleur Riley, Magdalena Harris, Hannah Louise Poulter, Helen J. Moore, Daniel Ahmed, Graham Towl, Tammi Walker

**Affiliations:** 1 Department of Psychology, Durham University, Durham, DH1 3LE England, UK; 2 Department of Public Health, Environments and Society, London School of Hygiene and Tropical Medicine, London, WC1E 7HT England, UK; 3 School of Social Sciences, Humanities and Law, Teesside University, Middlesbrough, TS1 3BX England, UK; 4 Foundations Medical Practice, Acklam Road, Middlesbrough, TS5 4EQ England, UK

**Keywords:** Harm reduction, People who inject drugs, Heroin-Assisted Treatment, Opioid dependency, Injectable opioid therapy, Recovery

## Abstract

**Background:**

Heroin-Assisted Treatment (HAT) is well evidenced internationally to improve health and social outcomes for people dependent on opioids who have not been helped by traditional treatment options. Despite this evidence base, England has been slow to implement HAT. The first service outside of a trial setting opened in 2019, providing twice-daily supervised injections of medical-grade heroin (diamorphine) to a select sample of high-risk heroin users in Middlesbrough. This paper explores their experiences, including the negotiation of the strict regularly controls required of a novel intervention in the UK context.

**Methods:**

We conducted in-depth interviews with service providers and users of the Middlesbrough HAT service between September and November 2021. Data from each group were thematically analysed and reported separately. This paper details the experiences of the twelve heroin dependent men and women accessing HAT.

**Results:**

Participants’ accounts of HAT treatment evidenced a tension between the regulatory constraints and uncertainty of treatment provision, and the positive outcomes experienced through supportive service provision and an injectable treatment option. Limited confidence was held in treatment efficacy, longevity of funding, and personal capacity for treatment success. This was counteracted by a strong motivation to cease engagement with the illicit drug market. While attendance requirements placed restrictions on daily activities, participants also experienced benefits from strong, supportive bonds built with the service providers through their continued engagement.

**Conclusions:**

The Middlesbrough HAT programme provided benefits to a high-risk population of opioid dependent people who were unable or disinclined to participate in conventional opioid substitution treatments. The findings in this paper highlight the potential for service modifications to further enhance engagement. The closure of this programme in 2022 prohibits this opportunity for the Middlesbrough community, but holds potential to inform advocacy and innovation for future HAT interventions in England.

## Background

Britain has the highest reported opioid using population in Europe [1, 2]. Drug-related deaths (DRDs) in England and Wales are at a record high, increasing by 60% between 2010 and 2020 [3]. Opioids (such as morphine and heroin) were implicated in almost half of DRDs (49.6%) with the highest prevalence among men aged 40–50 years [1, 3]. Increased opioid-related deaths have been attributed to increasing polydrug use, in particular concurrent use of benzodiazapines and gabapentanoids in combination with opioids [4–6]; co-morbidities and delayed health care access among an ageing opioid using population [7, 8]; and decreased government funding for drug treatments and services [9]. The burden of DRD is particularly evident in areas of high geographical deprivation, with a DRD prevalence in the North-East of England three times that of London [3]. In the North-East town of Middlesbrough, one of the most poorly resourced areas in England [10], citizens are statistically more likely to die from a DRD than a car accident [11]. In a context of high levels of deprivation and stretched social service provision, this increase in drug-related deaths among the most marginalised calls for an urgent and innovative drug treatment and policy response [12].

### Drug treatment provision in England and Wales

Although harm reduction policy and practice differ across UK countries, drug treatment policy in England and Wales has shifted over the past three decades from a harm reductionist response towards an abstinence-based model of service provision [13–16]. The former approach aims to reduce health and social harms associated with the criminalised use of substances, such as blood-borne virus acquisition, criminal engagement and fatal overdose, without expectation of drug use cessation [13–15]. Injecting equipment provision and maintenance opioid substitution treatment (OST), such as methadone and burprenorphine, fall under this remit [16, 17]. Support for maintenance-oriented OST throughout the 1990s diminished in the following decade, with the 2010 UK Drug Strategy [18] enscribing abstinence as the primary goal of drug treatment engagement. Increasing rates of DRD in the decade since 2012 [17] resulted in calls from leading academics for government policy to include harm reductionist responses to DRDs, including heroin-assisted treatment [17, 19, 20]. These have not been realised in the latest Drug Strategy ‘From Harm to Hope’ [21], which makes little explicit reference to harm reduction [19], continues to define substance dependence in criminological rather than public health terms [22], and advocates a ‘drug-free’ life for people with substance dependency [19]. Thus, the Westminster government remains committed to an abstinence-orientated model of service provision across England and Wales, despite rising DRDs among the most vulnerable and the failure of current drug treatment services and policies to stem this public health crisis.

Standard treatment for opioid dependency in the UK is methadone or buprenorphine OST, commonly administered through pharmacies on a daily supervised basis through to weekly take-home doses. These treatment modalities have a strong evidence base for reducing drug-related health harms and illicit drug market engagement [14, 15, 20]. They do, however, not suit all opioid dependent individuals, with an estimated 46% of the population not engaging with OST [23, 24] and many maintaining illicit heroin use while in treatment [25, 26]. Barriers such as limited accessibility, stigmatisation, and high-intensity treatment regimes can impede engagement and retention, leaving many individuals cycling in and out of treatment [23, 24, 27]. A significant population of opioid users remain in need of acceptable and accessible treatment options to address their increased risk of DRD, imprisonment, homelessness, and other health and social impacts related to illicit substance use [28]. This is particularly the case for long-term opioid users living in areas of high deprivation for whom OST has proved unhelpful.

### Heroin-Assisted Treatment: international evidence

Heroin-Assisted Treatment (HAT) is an innovative alternative to traditional forms of OST for long-term opioid dependency [29]. HAT consists of providing service users with injectable, synthetic grade heroin, known pharmaceutically as diamorphine/diacetylmorphine. HAT has been established in Switzerland since the mid-1990s and was subsequently adopted into standard practice in Canada, Germany, Holland, Denmark, and Luxembourg [29]. Evaluations of these services consistently demonstrate HAT as effective in reducing illicit heroin use, criminal engagement, risk of hepatitis infection and DRDs, and improving service users’ physical and mental wellbeing [29–36].

In the UK, Strang and colleagues conducted the Randomised Injecting Opiate Treatment Trial (RIOTT); [29, 37, 38]. RIOTT was conducted over a six-month period in three sites across the UK, including one site in the North-East of England, close to Middlesbrough. The RIOTT trial reported significantly decreased or discontinued illicit heroin use and improved treatment retention (compared to OST) as well as a general increase in positive psycho-social health and functioning outcomes among trial participants [29, 37, 38]. In January 2012, injectable opioid treatments became a recognised second line treatment in the UK, for “the small number of people who have repeatedly failed to respond to standard methadone treatment or residential rehabilitation” [39]. However, for reasons likely related to implementation barriers such as public safety concerns and financial cost [28] HAT did not become operational within the UK for a further seven years.

### Middlesbrough HAT (MHAT)

In 2019, a Middlesbrough drug and alcohol clinic became the first in the UK to offer HAT as fully regulated, non-trial treatment offer, followed closely by another clinic in Glasgow. HAT in Middlesbrough consisted of twice-daily diamorphine injections within an existing specialised clinic for drug and alcohol treatment. An initial quantitative evaluation of Middlesbrough HAT’s (MHAT) first year in operation reported a 60% reduction in service users’ criminality, significantly decreased street heroin use, reduced homelessness, and improved psychosocial wellbeing [4]. However, the impacts of treatment on service users’ lives and the mechanisms that promote these positive outcomes are less well understood.

The Middlesborough HAT service was carefully regulated, with a high-intensity delivery schedule, strict eligibility criteria and monitoring procedures designed to mitigate risk for both service users and the public [40]. Monitoring procedures included twice-daily clinic-based supervision during intravenous or intramuscular self-injection, weekly toxicity screening, and daily breathalysation for identified alcohol users. Additionally, methadone was prescribed to alleviate withdrawal symptoms between short-acting diamorphine doses, with methadone collection monitored through contact with the pharmacy. Such highly regulated and intensive drug treatment models have been criticised for restricting service users’ daily movements, constricting freedom, and compromising service users’ autonomy [40–42]. Furthermore, moving away from illicit drug use can comprise multiple challenges, with reports of social isolation, separating from entrenched social networks, and stigmatisation described as ‘pains’ impacting recovery outcomes [43, 44]. It is vital, therefore, to understand how HAT is experienced in a UK context, from the perspective of service users and in relation to these challenges, to inform recommendations for service modifications at scale.

### Heroin-Assisted Treatment—service users’ perspectives

Qualitative research exploring service user experiences of HAT is limited [41]. Romo et al. [45] conducted ethnographic research and interviews with HAT trial participants in Spain. In line with quantitative evidence, they reported decreased illicit drug use and criminal activity and emphasised the benefits of injectable diamorphine provision in decreasing service user stigmatisation and encouraging social engagement [45]. Boyd and colleagues have produced a body of qualitative research exploring the experiences of participants from Vancouver’s HAT trials [46–49]. Again, findings reported positive impacts of HAT on illicit substance use, crime, and health and emphasised the importance of staff relationships and collective identity in participants’ experiences [49]. These papers also highlighted ethical concerns about the discontinuation of HAT trials, reporting the negative impacts on participants and the importance of ethical exit strategies [46, 49]. Given the dearth of HAT service provision in the UK, there is limited local contextual evidence illustrating service users’ perspectives. This paper aims to address this gap, reporting analysis of qualitative data generated with Middlesbrough HAT service users, with a focus on the tensions experienced in initial treatment engagement and ongoing adherence.

## Methods

The findings reported here form part of a larger study funded by the National Institute of Health and Care Research. The study aimed to evaluate the Middlesbrough HAT service, focussing on recruitment, retention, and impact. We conducted in-depth interviews with staff, stakeholders and service users, and a small exploratory examination of service users’ health needs. The analyses presented here pertain specifically to data generated with HAT service users.

### Study setting

Middlesborough HAT opened in 2019, with capacity for up to fifteen service users. The clinic was situated within an existing specialised primary care service for drug and alcohol treatment, offering a range of drug and alcohol treatments such as OST. The HAT clinic was open 7 days a week, 365 days a year, between 8am and 6 pm. HAT service users were supervised within the clinic twice daily, for morning and afternoon doses of self-injected diamorphine, with a 4–5-h window between doses. Methadone was prescribed to mitigate withdrawal symptoms overnight, collected from a nearby pharmacy for self-administration at home. Diamorphine titration and dosing were monitored through weekly consultation with an on-site doctor. Daily injections were supervised by a nurse and health-care assistant, who monitored service users prior to and after each dose, reserving the right to refuse a dose if they had concerns about service user safety. Staff could also monitor methadone intake at their discretion, for example, if doses were not regularly collected. If three HAT doses were missed, the service user was required to recommence monitored re-titration under supervision of the prescribing doctor.

### Data generation

In-depth semi-structured interviews were conducted between 30/09/21 and 25/11/2021. Participants included all active HAT participants at the time of research (*N* = 10) plus two participants who were no longer in treatment. Clinic staff referred eligible participants to the researchers, who explained the study and provided an information sheet. Written informed consent was obtained by all participants prior to interview commencement. Interviews were conducted by two members of the research team (HP and FR) and were held in a private room at the HAT service to ensure privacy and confidentiality. The interviews were informed by a topic guide developed to assess different aspects of service recruitment, accessibility, treatment experiences, and impact. Participants were renumerated with a £20 voucher per interview. Predominantly gender-neutral pseudonyms were adopted to protect the anonymity of the small number of female service users. Ethical approval for the study was obtained from the Health Research Authority (HRA) and Health and Care Research Wales (HCRW) (IRAS ref: 292,909).

### Data analysis

Interviews were performed in English, recorded, and transcribed verbatim. Interview duration varied from 30 to 90 min, 60 min on average. Data were analysed through an iterative process guided by thematic analysis [50]. Transcripts were read through in detail by FR, with a proportion assigned open codes. Open codes were consolidated through team discussion to inform a coding framework against which all transcripts were coded. Second-level coding and analysis were developed through discussion with team members and senior researchers (TW, MH, HP) in an iterative process, leading to the generation of inductively derived analytic sub-themes.

### Participant characteristics

Twelve HAT participants were included in the study, nine men and three women, aged 24–55 (M = 42 years). The full cohort of HAT service users at the time of study were included, which constituted ten participants. Two further participants had ceased or completed treatment. Across the sample, treatment engagement length ranged from two weeks to two years, one month (M = 12 months).

#### Findings

Two primary themes were generated from the data. The first describes initiation tensions, explored against two sub-themes: (1) service users’ lack of confidence in treatment and doubts regarding their capacity for treatment success; (2) service users’ strong motivation for change overcoming initial hesitancy. The second primary theme describes adherence tensions, explored in relation to: (1) limited freedom and risk of unwanted social encounters due to twice-daily clinic visits, contrasting with (2) the development of strong bonds with staff and experiences of de-stigmatisation.

#### Theme 1: initiation tensions—lack of confidence versus readiness for change

This theme explores the tensions experienced by service users in relation to treatment engagement. The first of two sub-themes describes service users’ reticence to engage in treatment due to lacking confidence in both treatment efficacy and their own capacity for treatment success. This is contrasted in the second sub-theme which describes service users’ strong motivations for change and readiness for a novel treatment intervention.

### Lack of confidence in treatment efficacy and personal capacity for success

A general lack of trust and pessimism about drug treatment services and government-led health-care initiatives were expressed by many participants when discussing their willingness to engage with HAT. For example, one participant considered standard methadone treatment constraining, ‘holding’ service users indeterminately in treatment, echoing previous research in which methadone is likened to ‘liquid handcuffs’ [51].

*They offered me this, and I turned it down, just like that…….Because I thought it was going to be like a methadone programme, you get on it and you’ll be on it for years and years and years, you can’t get off it. – Billy*

Another participant, Georgie, expressed concerns about relinquishing control and autonomy over their own heroin consumption, transforming what they deemed as optional heroin use into a regulated ‘habit’ under state control. Participants’ pessimism and mistrust were likely rooted in their repeated experiences of ‘failed recovery’, which diminished trust and confidence in treatment efficacy [43, 52, 53]. Service users also expressed apprehension regarding treatment longevity due to concerns around funding.

*How long am I going to be funded for? What happens if the funding goes and then I’m left back on the streets? Am probably going to end up back in jail…..my future basically is in the hands of HAT. - Frankie*

Frankie’s anxiety about the potential repercussions of treatment cessation implies a sense of vulnerability in committing to a treatment programme with an uncertain future [54]. Such concerns were exacerbated by service users’ beliefs that a previous HAT trial in a nearby town was discontinued abruptly, leaving trial participants unsupported and subject to unpleasant withdrawal symptoms (or ‘rattling’).

*Because of what happened in [name of nearby town] where the funding got dropped and everyone just got left to rattle- where here I spoke to the staff and the staff said, “We get told six months in advance whether the funding’s going to get dropped so we’ve got six months to reduce you, so don’t ever worry.’’ – Charlie*

Other participants expressed a lack of confidence in their own capacity to succeed in treatment. Billie described their initial reaction to the treatment offer; ‘’I thought, there’s no way I can do that.’’ Thus, fear of failure and low self-efficacy created an initial reticence to engage in treatment [55]. Other participants suggested that MHAT’s eligibility criteria and monitoring regulations surrounding drug and alcohol use were off-putting, particularly for individuals who struggled with alcohol use and other substance dependencies.

*There is still a few people out there who I think would need help but they’re dead wary of it………. because they’ll have loads of addictions, tablets and something else or crack [cocaine] or they’re heavy drinkers……….I get breathalysed every day, so that’s why people say, I don’t want to bother, because there’s them type of hurdles there to get over. - Ray*

Thus, some individuals were deterred from engaging with treatment for opioid dependency for fear that multiple dependencies may render them ineligible for treatment, or incapable of meeting treatment requirements. This may serve to alienate potentially eligible service users who perceive their ‘hurdles’ as insurmountable, particularly if treatment eligibility requires a degree of mastery over multiple substance dependencies *prior* to treatment [55, 56]. Some participants experienced difficulties and delays commencing MHAT recruitment due to difficulties stabilising on methadone prior to treatment.

*I was out of [methadone] treatment a lot. So every month I was like out of treatment, twice in a month. So it became a bit of a problem to try and get me enrolled, because I hadn’t filled the criteria properly because of not taking my methadone on time. – Bobby*

This is particularly problematic given that, by definition, HAT’s target treatment population are long-term opioid dependent individuals who have repeatedly struggled to benefit from standard treatment offers such as methadone. Stability on methadone as a prerequisite for HAT created a somewhat counter-intuitive barrier for individuals who found stability on methadone difficult to achieve. This may serve to further marginalise or penalise the individuals for whom HAT may be most appropriate.

### Motivation for change and readiness for a novel approach

Despite some participants’ initial reticence surrounding HAT, all participants expressed a very strong desire and readiness for change, with many citing painful life events such as periods of incarceration, loss of relationships, health crises, and near-death experiences as motivators for engaging with HAT.

*I was in and out of hospital a lot with my breathing because of the heroin. I was injecting it. Injecting into my veins, so my veins were blocking the oxygen. And they got bad, really bad. I nearly died once. - Billy*

Such ‘turning points’, or experiences of hitting ‘rock bottom’, have been suggested as strong motivators for change, pushing individuals towards drug use cessation as the pains of continued drug-use come to outweigh the potential pains of leaving this lifestyle behind [43, 57]. Participants’ anxieties over physical health and mortality were acutely linked to the unpredictability of street heroin quality.

*It is really dangerous stuff, and you don’t know what you’re putting into yourself. Once could be alright, the next time you get it on the night it could be off someone else or a different batch or bashed, and that’s what’s the most dangerous thing. You don’t know what you’re getting. – Jacky*

Jacky describes the inherent dangers of street heroin use, reflecting the vulnerability and risk experienced by people dependent on an illicit and unregulated drug market. Thus, participants were motivated by the harm-reducing potential of MHAT, with medical diamorphine perceived as a safer and healthier choice than continued use of potentially contaminated and harmful street heroin. Alongside harm-avoidance, participants also expressed goals that aligned with broad definitions of ‘recovery’ [58, 59] such as improved social functioning and better quality of life [58, 59].

*I was so desperate to get on this course because I wanted to stop, I wanted to get off the merry-go-round……I want to be off everything….I want a clean, healthy life. I want to rebuild the relationships, and enjoy the rest of my life – Bobby*

Like Bobby, many participants expressed abstinence-based treatment-objectives, a desire to be ‘drug-free’, ‘get clean’ and ‘just to be a normal member of society’ (Franky, Jessy). While this may indicate the possible internalisation or expression of social norms and expectations surrounding drug treatment and recovery, participants nonetheless appeared to view MHAT as offering a pathway to not only harm-reduction and broader recovery-related goals, but also eventual abstinence.

The novelty of MHAT’s approach to opioid dependency was also appealing to participants. Many reflected on repeated ‘failed recovery’ attempts as motivators to try something new.

*It's like, you've had methadone in the past, that hasn't worked, you've had 12 step programmes in the past that hasn't worked, you had rehab in the past, that hasn't work, so why not try something that might work isn’t it? – Alex*

For Alex, and other participants, MHAT represented new hope and opportunity to achieve a broad range of harm-reduction and recovery-related goals. This illustrates the potential for innovative treatments to re-engage previously disengaged or disillusioned populations.

Here service users’ accounts display an initial reticence to engage with HAT due to lack of confidence in treatment efficacy and longevity, and a reluctance to concede control to a treatment programme with an uncertain future. Difficulties in stabilising prior to treatment created further barriers to recruitment. However, participants’ strong motivation to avoid harm and achieve recovery-related goals, and readiness to try a novel treatment, ultimately overcame initial anxieties related to commencing treatment.

### Theme 2: adherence tensions—restriction and risk versus social support and de-stigmatisation

This theme describes some of the negative and positive impacts of MHAT’s intensive treatment schedule on participants’ lives. The two sub-themes encapsulate service users’ difficulties with the restrictive twice-daily treatment schedule and risky contact with other people who use drugs***,*** and contrasting experiences of supportive staff relationships and de-stigmatisation in treatment.

#### Restrictions of twice-daily commitment and risk of contact with other people who use drugs

Some participants reported experiencing hardship due to the intensity of Middlesbrough HAT’s treatment schedule, specifically the twice-daily morning and afternoon visits to the clinic for supervised dose injection. The short four-to-five-hour window between doses restricted how participants could spend their days, proving particularly problematic for those who relied on public transport to access the service.

*I think the most annoying part of it is having to come twice a day and especially from where I live that’s the only thing…….I have to get.….four buses to get here and home and then four buses to get here and home again. So, I think eight buses a day – Jay*

Other participants described MHAT’s daily treatment schedule as ‘hardcore’ (Ray) and ‘a full-time commitment’ (Jacky). Frankie described his experience of treatment as follows; *‘you can’t do nothing because you’ve got to come here twice a day, it’s really hard, it’s really difficult’*. This supports previous criticisms of HAT as restrictive and constraining, or a form of social control that dictates service users’ daily movements and activities [42]. Consequently, participants relied on family and friends for assistance with transport, or filled time between doses with shopping, accessing wrap around support, visiting family or volunteering within the service.

*My routine is I get up, I take one Zopiclone. I come here, have my HAT, go do whatever I need to go do and then I come back at 2 o’clock, have my HAT and then go home—Frankie*

Further tension was experienced by participants due to the services’ co-location within a community-based drug treatment centre. Unwanted encounters with active drug users visiting the clinic, predominantly outside the building and in the shared waiting room, were felt to compromise service users’ privacy and anonymity (Billy, Jacky). For others, contact with active drug users increased the risk of illicit drug use and relapse.

*Because I’m on HAT, the downfall was, is I was bumping into people you know because of the place where I’m coming to, so I’m bumping into people and going with them…… I’ve relapsed about three times in the first year…because of the tablets I found myself in a couple of dodgy places. And … I made the mistake of using gear. - Bobby*

Contact with active drug users was especially problematic for MHAT service users who struggled with multiple dependencies and poly-drug use. A small number of participants spoke of the intermittent use of street tablets, illicit versions of prescription drugs such as benzodiazapines and zopliclone. Illicit street tablets are unpredictable in quality, strength, and effect [5, 60] and can be dangerous when taken alongside diamorphine, leading to some incidences of sedation and subsequent dose refusal.

*I was in a safe environment, they were looking out for me, I turned up a couple of times wrecked, not all the time, but a couple of times wrecked and they turned me away; rightly so, I would have died in there if they didn't. – Alex*

Participant suggestions for decreasing the risk of unwanted social encounters and poly-drug use included separate waiting spaces for MHAT service users (Billy) and the introduction of a third (evening) diamorphine dose to mitigate illicit self-medication overnight (Georgie). Some participants suggested that incorporating treatment for multiple substances into the HAT provision would better address risks and issues associated with multiple dependencies and encounters with active drug-users.

*I think they [the service providers] should step up……If you’re addicted to something, it’s a place for an addiction, it should be addressed. – Georgie*

Overall, service users reported some negative impacts of MHAT’s high intensity delivery schedule and co-location within a community-based drug service. Twice-daily clinic attendance restricted participants’ freedom of daily activity and increased the likelihood of unwanted contact with individuals engaged with the illicit drug market. This in turn increased the risk of relapse or poly-drug use, and was experienced as both difficult and undesirable by service users.

#### Supportive staff relationships and de-stigmatisation

Not all consequences of MHAT’s intensive treatment schedule were negative. Twice-daily clinic attendance facilitated the development of very close relationships with staff and other MHAT service users, creating a tight-knit supportive community within the clinic.

*I’ve got a good bond with the staff in here and in HAT it’s different, like you get closer with the staff. Like me and [name of staff] go on as if we’re sisters, that’s how close our bond is now I’m on HAT. - Charlie*

For Charlie, MHAT service users represented a ‘special’ group of service users, whose bonds with staff exceeded regular staff/service user relationships, and those of non-HAT service users. Others described always having ‘someone to talk to’(Billy), and staff and MHAT service users being ‘like a big family’ (Frankie). Regular clinic visits, therefore, facilitated the formation of a collective identity within a supportive community environment [15, 45]. Furthermore, participants reported increased engagement with other health and social care services available within the clinic, due to already being at the clinic for their HAT dose. Access to wrap-around services and growing commitment to treatment were facilitated by high levels of staff investment in MHAT service users’ wellbeing.

*They was constantly in touch ……make sure I was always getting to the appointments all the time and stuff…… They helped me want it. They showed me that I did want it by being the way they were being. – Alex*

Staff relationships and investment became external motivators for continued treatment engagement, despite its challenges. Some participants did, however, report feelings of apprehension around supervised injecting. Participants described the highly stigmatised activity of injecting heroin [61] as ‘private’, ‘personal’ and ‘intimate’ (Jessy, Sam, Jacky). Fear of stigmatisation and judgement was reflected in one participants’ expression of discomfort and exposure during supervised injecting early in treatment.

W*hen the girls were sat there watching and I was like, “Look, you’ve got to stop looking at me’’ but they said, “We’ve got to watch you” and I learnt to grow into it anyhow so I learnt to accept it……But it was difficult yes I was like all eyes on me sort of thing……. It’s degrading, it’s like a normal person watching you digging heroin you know what I mean? – Georgie*

Despite some initial discomfort and shame during supervised injecting, participants overwhelmingly reported a rapid transition to feelings of comfort, safety and acceptance within the treatment room. One participant stated; ‘’*there was a comfort in it like I didn’t feel judged.’’* (Alex). This transition was largely facilitated by staff’s sensitive and respectful approach to supervision.

*They’re really nice in there. They let you get on with it. They don’t, they watch you to make sure that you’re not hurting yourself or you’re not going in places that you shouldn’t be…..so they do watch over you but they don’t crowd you or they don’t stand over you a lot or anything. – Jacky*

Respectful treatment by staff served to destigmatise injecting behaviours for participants and mitigate feelings of shame and judgement. For many, this de-stigmatisation extended beyond the treatment room to a more holistic sense of acceptance of the participants as individuals. Participant Sam described being in the treatment clinic; *‘’I felt good. I feel people see me for what I am, and there’s nothing wrong with that’’.*

Thus, participants experienced positive, de-stigmatising effects of regular clinic attendance, while simultaneously experiencing constraints and limitations on freedom. In balancing this tension, participants reported engaging in intuitive cost/benefit analyses, where the difficulties of the high-intensity treatment, or the ‘pains of recovery’ [43] were compared to the predicted pains of recommencing street heroin use. As such, participants justified the sacrifices and difficulties involved in twice-daily community-based treatment as ultimately preferable to the dangerous and stigmatised activities involved in street heroin acquisition.

*I don’t mind because coming twice a day is better than having to do all the stuff I was doing all day. It takes more time to score twice than it would to walk here twice a day. I don’t miss begging because I hated that. I don’t miss shoplifting. I don’t miss going on the beat. – Charlie*

Overall, while being restrictive of participants’ freedom and increasing risk of contact with illicit drug-users, twice-daily clinic visits also facilitated the development of close, supportive staff and peer bonds. Furthermore, regular clinic visits also facilitated increased engagement with other psycho-social services available to service users, and monitored injecting offered an opportunity for de-stigmatisation of drug-using practices within a clinical setting, promoting feelings of acceptance and belonging.

## Discussion

This paper explored Middlesbrough HAT service users’ experiences of treatment, with particular focus on tensions experienced around treatment initiation and ongoing treatment adherence. Participants experienced internal conflict during initial recruitment due to negative preconceptions of drug-treatment services after repeated unsuccessful attempts at treatment. As MHAT participants are, by definition, long-term opioid users who have found standard treatment unhelpful, it is important to consider ways to build trust with individuals who may feel disillusioned with drug-treatment services. Peer support and treatment ‘champions’ may be one way to address this need [15, 62].

Anxieties about funding insecurity impacted treatment desirability and engagement, with service users’ concerns founded in the known uncertainty of MHAT funding and the potential personal impact of service discontinuation. In November 2022, after three years in operation and approximately a year after this study was conducted, service users’ concerns were validated when MHAT funding was stopped and the service closed [63, 64]. While the impact of treatment cessation on MHAT service users is currently unknown, evidence from discontinued HAT trials in Canada [49] and Belgium [65] suggests adverse effects of involuntary HAT cessation. These included a return to street heroin use, increased risk of DRD, and a deterioration of the benefits accrued over the course of treatment [49, 65]. This raises important questions about the ethics of discontinuing established medical care for particularly vulnerable, high-risk individuals. Given the evidenced benefits of maintenance treatment [13–15] and the potential consequences of treatment cessation [49, 65], HAT should be considered a long-term treatment solution for opioid dependency, with ongoing service user needs and input paramount to funding decisions. Long-term or permanent funding would enable HAT service users to undergo treatment with a secure expectation of continued care.

Service users’ lack of confidence extended to their personal capacity to achieve treatment eligibility or success, particularly in individuals who struggled to achieve adequate stability or mastery over multiple substances prior to treatment. The somewhat counter-intuitive requirement for individuals to prove stable on methadone *prior* to MHAT commencement can marginalise individuals for whom HAT may be most beneficial. Greater flexibility in eligibility criteria, regulatory procedures, and transitional support may improve treatment desirability and better facilitate recruitment of suitable service users.

Participants were ultimately motivated to overcome initial treatment scepticism by a strong desire for change, driven by worsening physical health, concerns about mortality, periods of incarceration, and other painful life experiences. Described in recovery literature as ‘rock bottom’ experiences [57], these instances acted as motivators for change, pushing service users into treatment despite their apprehension. Thus, barriers were overcome and treatment initiated when perceived negative consequences of illicit drug use outweighed negative preconceptions of treatment. Importantly, most participants were motivated by both harm-reduction and abstinence-focused treatment goals, opposing dyadic conceptualisations of drug treatment outcomes as either maintenance or abstinence based [59, 66]. Reported motivations included improved health, wellbeing and social functioning, a desire for a ‘normal’ life, as well as illicit drug consumption reduction. Such motivations sit within broad definition recovery as a multi-faceted, dynamic process of change across multiple life domains including improved physiological, psychological and social functioning, where abstinence is not a prerequisite for recovery [44, 58, 67–70]. Thus, HAT appeals to individuals who are motivated by a broad range of recovery-related goals, including but not limited to reduced substance use.

Service users experienced further tensions relating to MHAT’s high-intensity delivery schedule. Twice-daily, clinic-based supervised injections often restricted participants’ daily movements, limiting choice, autonomy and freedom. Employment, volunteering, education, and holidays are rendered effectively impossible while undergoing twice-daily supervised treatment. As active participation in and contribution to community and society are key components of recovery [44, 69, 70], twice-daily clinic attendance may constrain MHAT’s therapeutic potential by limiting service users’ opportunities for active citizenship [40–42]. Participants’ desire to regain freedom and autonomy may not only encourage disengagement, but inadvertently push participants *through* treatment towards the ‘end-goal’ of abstinence, potentially undermining the value of harm reduction and other broader recovery-related treatment outcomes [15].

However, despite exerting constraining effects on participants’ freedom, twice-daily clinic visits also facilitated the development of close bonds with staff and fellow service users. Further, the destigmatising effects of medicalised injecting fostered a sense of acceptance for participants within the clinic environment, contributing to a collective sense of community and support within the small cohort of MHAT service users. Positive social connection is a catalyst for increased well-being, self-efficacy, and hope, which in turn promotes increased identification and engagement with positive social groups and strengthens the groups’ sense of pride in their collective identity [67, 69]. Service users’ growing sense of belonging improved self-worth and increased self-efficacy were inter-connected with their identification as members of a ‘special’ group of service users within the Middlesbrough clinic, demonstrating how personal recovery is a relational, dynamic, and socially embedded process [70, 71]. Thus, while the regularity of interaction with staff and peers likely intensified and accelerated the development of positive personal and collective identities, contributing to global improvements in service users’ wellbeing, twice-daily treatment simultaneously curtailed service users’ wider community engagement.

Participants experienced further tension due to the co-location of MHAT within an existing community-based drug treatment service. While co-location improved access to health care and other psychological and social services, it also increased the likelihood of unwanted contact with individuals active in the illicit drug market. Contact with active drug users, particularly those known to service users, may complicate the process of identity transformation described above, presenting conflict between old and new social identities [70]. This could be mitigated by separate entrances or waiting spaces for HAT service users, increasing privacy and perhaps further strengthening group cohesion.

Various tensions experienced by MHAT service users described in this paper could be mitigated with greater flexibility in treatment delivery protocols. For example, providing take-home afternoon doses for stable service users would increase participants’ daily liberty, while maintaining regular contact. Take-home doses of diamorphine are legally available within the UK [72] and offered in other HAT programmes internationally. The Swiss HAT programme [73] recently reported increased take-home doses of oral diamorphine during covid, reporting no adverse effects on treatment or security, and increased treatment retention, service user satisfaction and quality of life. Potentially then, flexible treatment alternatives may optimise service users’ experiences of treatment and recovery by increasing opportunities for active citizenship. HAT treatment policy should prioritise strategies that alleviate the negative consequences of high-intensity, community-based treatment while optimising the positive impacts of de-stigmatisation and supportive social interaction. Nonetheless, MHAT demonstrates the potential for innovative harm-reduction drug services to facilitate broad personal and social recovery experiences within a community setting.

## Limitations

This study’s small sample consisted of ten current HAT service users, one service user who had completed treatment (to attend rehab) and one who had withdrawn. Of the active MHAT service users, four had been in treatment for less than a month. While this was helpful in understanding the motivators, recruitment facilitators and early experiences of MHAT service users, experiences of long-term benefits were garnered from a relatively small cohort of six service users. Furthermore, interviews all took place on site within a private room in the clinic immediately post-dose. While participants were reassured of confidentiality and the impartiality of the researchers, interviewing within the service may have influenced participants to report an excessively positive perspective of the programme. Finally, the research team experienced difficulty in recruiting individuals who were offered but had refused MHAT, and service users who had discontinued treatment. Thus, results are primarily informed by service users who had ultimately overcome or worked with the reported tensions in recruitment and engagement. Future research could benefit from peer researcher involvement [74] to aid recruitment of individuals who have refused or discontinued treatment.

## Conclusion

This research explored the treatment experiences of service users engaged in Heroin-Assisted Treatment (HAT) in Middlesbrough. This high-risk group of individuals, for whom traditional OST regimes had consistently proved unhelpful, reported social, health, and personal self-efficacy-related benefits, despite the challenges of treatment engagement. Initiating treatment required participants to overcome a range of insecurities and doubts, both in the adequacy and security of the treatment offer and participants’ personal capacity for success. Anxieties were overcome by service users’ strong motivations for change and readiness for novel treatment solutions. The treatment schedule and location of the clinic also presented challenges, restricting freedom and increasing the risk of unwanted social contact. These challenges were offset by the development of strong staff and peer bonds, a sense of collective identity and increased self-efficacy which encouraged continued engagement. Findings illustrate the conflicting benefits and drawbacks of high intensity, community-based treatment engagement for a high-risk population and highlight the potential for service modifications to further enhance engagement. The closure of the MHAT programme in 2022 prohibits this opportunity for the Middlesbrough community, but holds potential to inform advocacy and innovation for future HAT interventions in England and elsewhere.

## Data Availability

The interview transcripts generated during the interviews in this study are not publicly available to preserve the confidentiality of the participants.

## Abbreviations

DRD: Drug-related death
HAT: Heroin-Assisted Treatment
MHAT: Middlesbrough Heroin-Assisted Treatment
OST: Opioid substitution therapy
